# Supplementary material for: Inhibition of Protease-Activated Receptor-2 Activation in Parkinson’s Disease Using 1-Piperidin Propionic Acid
Source: Biomedicines. 2024 Jul 22;12(7):1623. doi: 10.3390/biomedicines12071623 (PMC11274518; doi:10.3390/biomedicines12071623)
Supplement: Supplementary file 1 [file biomedicines-12-01623-s001.zip › biomedicines-3113329-SI.pdf]

Supplemental Table S1. List and sequences of murine and human primers used for quantitative real time PCR.

**Gene**	**Primers**
IL-1β (murine gene)	(FW) 5’ GAAATGCCACCTTTTGACA 3’<br>(RV) 5’ TTGGAAGCAGCCCTTCATCTT 3’
TNF-α (murine gene)	(FW) 5’ ACGGCATGGATCTCAAAGAC 3’<br>(RV) 5’ GTGGGTGAGGAGCACGTAGT 3’
IL-6 (murine gene)	(FW) 5’ CCGGAGAGGAGACTTCACAG 3’<br>(RV) 5’ TGGTCTTGGTCCTTAGCCAC 3’
PAR-2 (murine gene)	(FW) 5’ CTGCTGGGAGGTATCACCCTT 3’<br>(RV) 5’ TTTCCCAGTGATTGGAGGCTG 3’
β-Actin (murine gene)	(FW) 5’ AGCCATGTACGTAGCCATCC 3’<br>(RV) 5’ CTCTCAGCTGTGGTGGTGAA 3’
SB3 (human gene)	(FW) 5’ GCAAATGCTCCAGAAGAAAG 3’<br>(RV) 5’ CGAGGCAAAATGAAAAGATG 3’
PAR-2 (human gene)	(FW) 5’ GCTAGCAGCCTCTCTCTCCT 3’<br>(RV) 5’ GTGGGATGTGCCATCAACCT 3’
β-Actin (human gene)	(FW) 5’ AGAGCTACGAGCTGCCTGAC 3’<br>(RV) 5’ GGATGCCACAGGACTCCA 3’
